# Supplementary material for: Early detection of chronic hepatitis B and risk factor assessment in Turkish migrants, Middle Limburg, Belgium
Source: PLoS One. 2020 Jul 27;15(7):e0234740. doi: 10.1371/journal.pone.0234740 (PMC7384618; doi:10.1371/journal.pone.0234740)
Supplement: S1 Fig — (PDF) [file pone.0234740.s001.pdf]

Code: HBVTR-……………..-……………. (in te vullen door de arts)

Voornaam:…………………………………..
Naam: ………………………………………..
Geboortedatum:

□□/□□/□□□□

(DD/MM/JJJJ)
Geslacht: man
Adres:
…………………………………………………
…………………………………………………

Naam (en voornaam) huisarts:
…………………………………………………
…………………………………………………
…………………………………………………

Plaats huisarts:……………………….................
…………………………………………………

VRAGENLIJST

A. Demografische gegevens

1. Wat is uw geboorteland?
   - □ België → ga naar vraag **3**
   - □ Turkije → ga naar vraag **2**
   - □ Ander land → ga naar vraag **3**

2. Indien u geboren bent in Turkije, wat was het jaar waarop u naar België kwam? (bv. in het jaar 1999)

   □□□□

3. Kruis aan wie van uw ouders geboren is in Turkije? (meerdere opties mogelijk)
   - □ Vader → beantwoord vraag **4**
   - □ Moeder → beantwoord vraag **5**
   - □ Geen van beiden → sla vraag **4 en 5** over

4. In welke regio is uw vader geboren ? (zie figuur)

□ Regio A: Marmara □ Regio B: Ege □ Regio C: Akdeniz
□ Regio D: İç Anadolu □ Regio E: Karadeniz □ Regio F: Doğu Anadolu
□ Regio G: Güneydoğu Anadolu

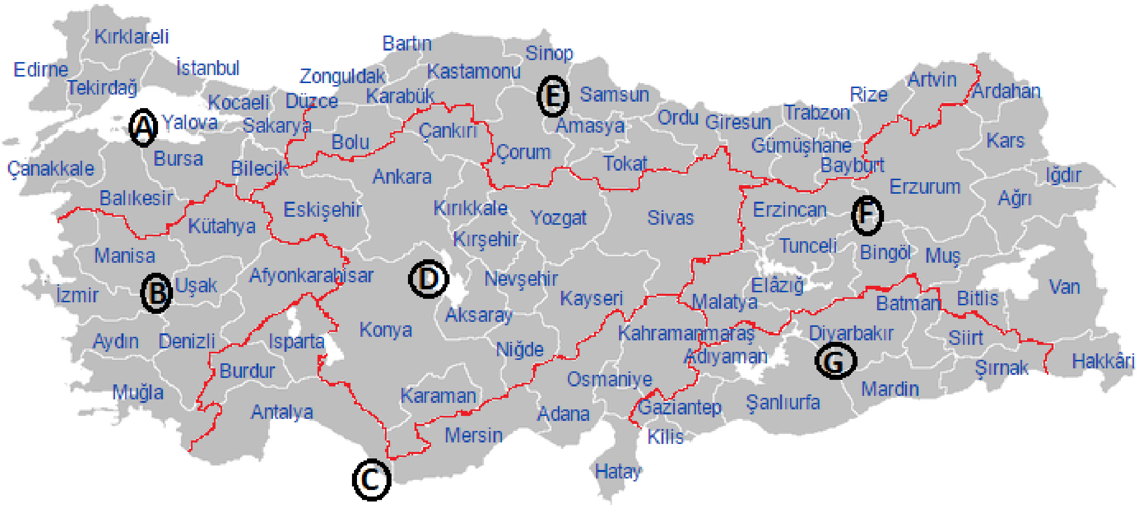

5. In welke regio is uw moeder geboren ? (zie figuur)

□ Regio A: Marmara □ Regio B: Ege □ Regio C: Akdeniz
□ Regio D: İç Anadolu □ Regio E: Karadeniz □ Regio F: Doğu Anadolu
□ Regio G: Güneydoğu Anadolu

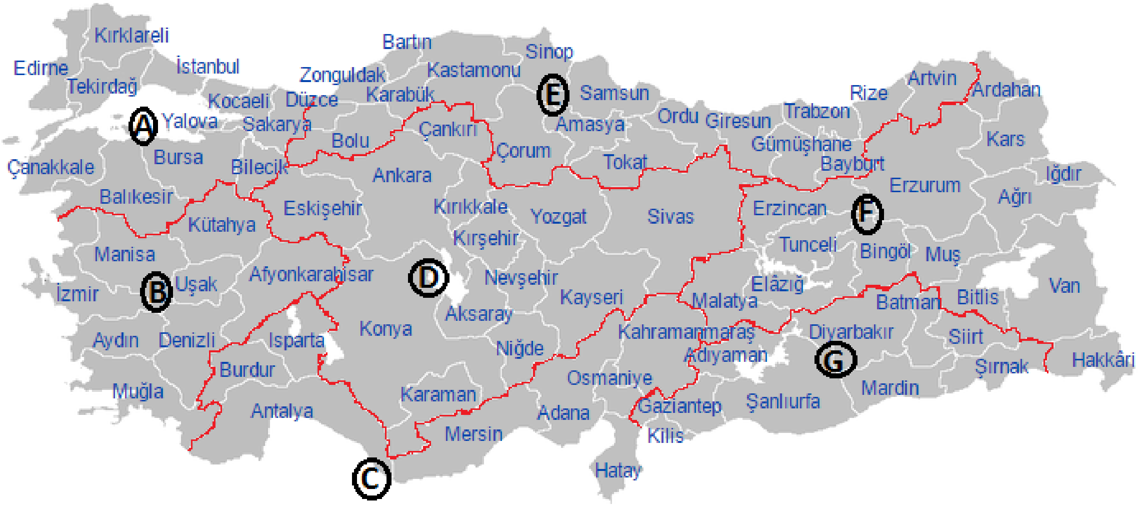

B. Besnijdenis

1. Bent u besneden geweest?
   □ Ja → ga naar vraag **2**
   □ Nee → sla vraag **2** en **3** over

2. Hoe bent u besneden geweest?
   □ Ik werd als enige besneden en dus niet gezamenlijk in een groep
   □ We werden gezamenlijk in een groep besneden
   □ Weet ik niet

3. Door wie bent u besneden geweest?
   □ De besnijdenis werd uitgevoerd door een arts
   □ De besnijdenis werd niet door een arts uitgevoerd
   □ Weet ik niet

C. Gezondheidszorg

1. Heeft u ooit bloed ontvangen? (meerdere opties mogelijk):
   □ Ja, ik heb voor 1972 bloed ontvangen in Turkije
   □ Ja, ik heb voor 1972 bloed ontvangen in België
   □ Ja, ik heb na 1972 bloed ontvangen
   □ Nee, ik heb geen bloed ontvangen

2. Bent u vroeger behandeld bij de tandarts in Turkije?
   □ Ja
   □ Nee

3. Heeft u in het verleden een operatie ondergaan? (meerdere opties mogelijk):
   □ Ja, ik heb in het verleden een operatie ondergaan in Turkije
   □ Ja, ik heb in het verleden een operatie ondergaan in België
   □ Nee, ik heb geen operatie ondergaan
   □ Geen van bovenstaande antwoorden

4. Heeft u in het verleden een behandeling met naalden (vb. accupunctuur, infuus) gekregen? (meerdere opties mogelijk):
   □ Ja, ik heb een behandeling met naalden gekregen in Turkije
   □ Ja, ik heb een behandeling met naalden gekregen in België
   □ Nee, ik heb geen behandeling met naalden gekregen
   □ Geen van bovenstaande antwoord

D. Gezin

1. Kruis aan wie van uw gezin hepatitis B virus infectie heeft (meerdere opties mogelijk):
   - □ Moeder
   - □ Vader
   - □ Ik heb een hepatitis B virus infectie
   - □ Andere familielid (vb. oom, tante)
   - □ Geen van bovenstaande antwoorden
   - □ Broer(s)
   - □ Zus(sen)
   - □ Echtgenote of vaste partner
   - □ Weet ik niet

2. Heeft u ooit een tandenborstel gedeeld met iemand uit het gezin?
   - □ Ja, meermaals (wetende en/of per ongeluk)
   - □ Ja, éénmalig (wetende en/of per ongeluk)
   - □ Nee

3. Heeft u ooit een nagelknipper gedeeld met iemand uit het gezin?
   - □ Ja
   - □ Nee

4. Heeft u ooit een scheermes gedeeld met iemand uit het gezin?
   - □ Ja
   - □ Nee

5. Heeft u ooit een gebruikte handdoek gedeeld met iemand uit het gezin?
   - □ Ja
   - □ Nee

6. Heeft u ooit uit hetzelfde bord gegeten met iemand uit het gezin?
   - □ Ja
   - □ Nee

E. Overige

1. Kruis aan wat van toepassing is bij u (meerdere opties mogelijk):
   □ Ik heb een tatoeage, bodypiercing of gaatjes in het oor laten zetten in Turkije
   □ Ik heb een tatoeage, bodypiercing of gaatjes in het oor laten zetten in België
   □ Ik heb geen tatoeage, bodypiercing of gaatjes in het oor laten zetten
   □ Geen van bovenstaande antwoorden

2. Heeft u vroeger een voetbehandeling met visjes (Fish spa) gekregen?
   □ Ja, ik heb vroeger een voetbehandeling met visjes gekregen in Turkije
   □ Ja, ik heb vroeger een voetbehandeling met visjes gekregen in een ander land dan Turkije
   □ Nee, ik heb geen voetbehandeling met visjes gekregen

3. Wat is het hoogst behaalde diploma van uw vader?
   □ Geen diploma
   □ Lagere school (basisonderwijs)
   □ Middelbare school (secundair onderwijs)
   □ Hogeschool of universiteit (hoger onderwijs)

4. Wat is het hoogst behaalde diploma van uw moeder?
   □ Geen diploma
   □ Lagere school (basisonderwijs)
   □ Middelbare school (secundair onderwijs)
   □ Hogeschool of universiteit (hoger onderwijs)

5. Bent u gevaccineerd tegen hepatitis B?
   □ Ja □ Nee □ Weet ik niet

   →Indien **Ja**, hoeveel inentingen heeft u gehad?
   □ 1 □ 2 □ $\geq$3 □ Weet ik niet

   →Indien **Nee**, waarom bent u niet gevaccineerd tegen het hepatitis B virus? (meerdere opties mogelijk)
   □ Ik hoef niet gevaccineerd te worden omdat ik me goed voel
   □ Ik had geen kennis over de mogelijkheid tot hepatitis B vaccinatie
   □ Ik moet zelf betalen voor de vaccinatie. Vaccin is te duur
   □ Onbekend, ik weet het niet
