# Supplementary material for: Early detection of chronic hepatitis B and risk factor assessment in Turkish migrants, Middle Limburg, Belgium
Source: PLoS One. 2020 Jul 27;15(7):e0234740. doi: 10.1371/journal.pone.0234740 (PMC7384618; doi:10.1371/journal.pone.0234740)
Supplement: S2 Fig — (PDF) [file pone.0234740.s002.pdf]

Kod: HBVTR-...............-.................(doktor tarafından doldurulacaktır)

Ad: ....
Soyad: ....
Doğum tarihi:

□□/□□/□□□□

(Gün/Ay/Yıl)
Cinsiyet: erkek
Adres:
....
....

Ev doktorunun adı (ve soyadı):
....
....
....

Ev doktorunun adresi:..
....

SORU LISTESI

A. Demografik bilgiler

1. Doğduğunuz ülke?
   □ Belçika → soru **3** e geçin
   □ Türkiye → soru **2** e geçin
   □ Başka bir ülke → soru **3** e geçin

2. Eğer Türkiye doğumluysanız, aşağı yukarı Belçikaya geldiğiniz tarih nedir? (örneğin sene 1999)

   □□□□

3. Baba ve annenin arasında kim Türkiye doğumlu? (birkaç seçenek mümkün olabilir)
   □ Baba → **4** üncü soruyu cevaplayınnız
   □ Anne → **5** inci soruyu cevaplayınız
   □ Hiçbiri → soru 4 ve 5 i atlayın, **B sünnet bölümüne** geçin

Kod: HBVTR-...............-.................(doktor tarafından doldurulacaktır)

4. Babanız hangi bölgede doğdu? Aşağıdaki haritaya bakınız.
   - □ A: Marmara bölgesi
   - □ B: Ege bölgesi
   - □ C: Akdeniz bölgesi
   - □ D: İç Anadolu bölgesi
   - □ E: Karadeniz bölgesi
   - □ F: Doğu Anadolu bölgesi
   - □ G: Güneydoğu Anadolu bölgesi

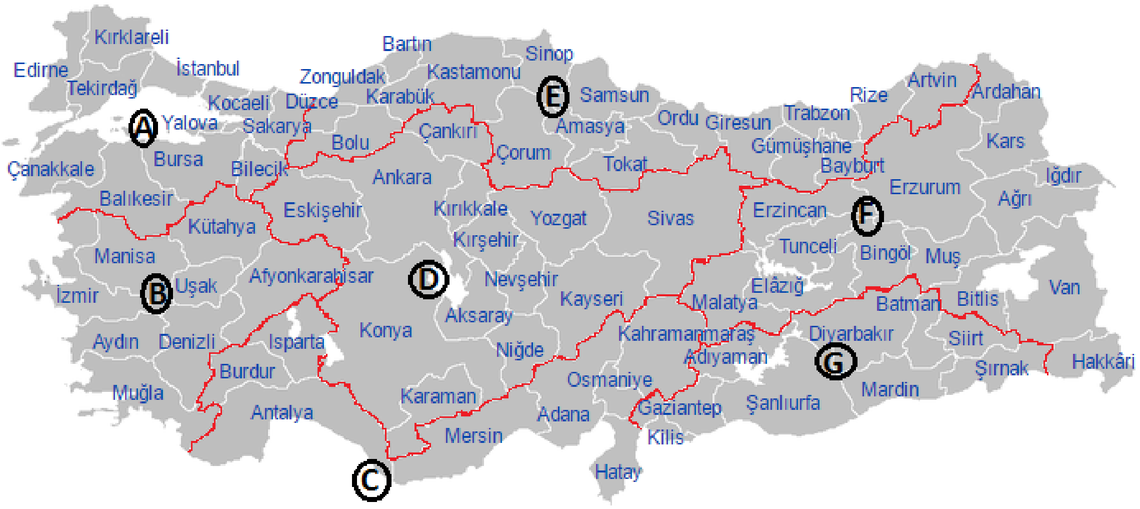

5. Anneniz hangi bölgede doğdu? Aşağıdaki haritaya bakınız.
   - □ A: Marmara bölgesi
   - □ B: Ege bölgesi
   - □ C: Akdeniz bölgesi
   - □ D: İç Anadolu bölgesi
   - □ E: Karadeniz bölgesi
   - □ F: Doğu Anadolu bölgesi
   - □ G: Güneydoğu Anadolu bölgesi

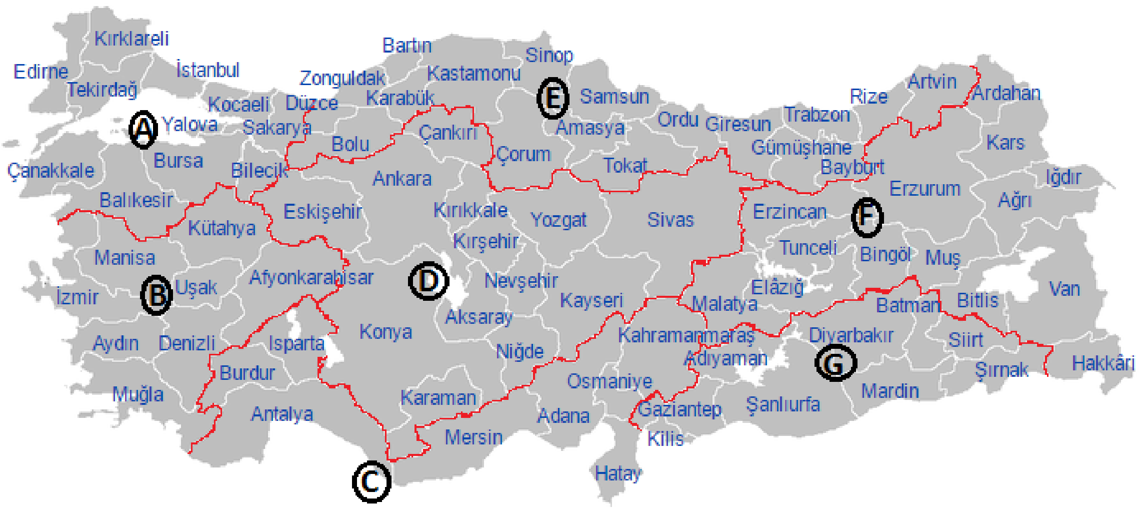

B. Sünnet

1. Sünnet oldunuz mu?
   □ Evet → soru **2** e geçin
   □ Hayır → soru 3 ve 4 ü atlayın, **C sağlık sektörü bölümüne** geçin

2. Toplu veya tek olarak mı sünnet edildiniz?
   □ Toplu olarak değil, tek olarak sünnet edildim
   □ Toplu olarak sünnet edildim (örneğin toplu olarak yapılan sünnet töreni ile)
   □ Bilmiyorum

3. Sünnet kimin tarafından yapıldı?
   □ Sünnet doktor tarafından yapıldı
   □ Sünnet doktor tarafından yapılmadı
   □ Bilmiyorum

C. Sağlık sektörü

1. Size kan verildi mi? (birkaç seçenek mümkün olabilir):
   □ Evet, bana 1972 yılından önce Belçikada kan verildi
   □ Evet, bana 1972 yılından önce Türkiyede kan verildi
   □ Evet, bana 1972 yılından sonra kan verildi
   □ Hayır, bana kan verilmedi

2. Türkiyede dişcide diş tedavisi gördünüz mü?
   □ Evet
   □ Hayır

3. Hiç ameliyat oldunuz mu? (birkaç seçenek mümkün olabilir):
   □ Evet, Türkiyede ameliyat oldum
   □ Evet, Belçikada ameliyat oldum
   □ Hayır, hiç ameliyat olmadım
   □ Üst tarafta yazılı olan cevapların hiçbiri

4. Önceden iğnelerle tedavi (örneğin akupunktur, enfüs) gördünüz mü? (birkaç seçenek mümkün olabilir):
   □ Evet, Türkiyede iğnelerle tedavi gördüm
   □ Evet, Belçikada iğnelerle tedavi gördüm
   □ Hayır, ben iğnelerle tedavi görmedim
   □ Üst tarafta yazılı olan cevapların hiçbiri

D. Aile

1. Ailenizde hepatit B virüs enfeksiyonu olan kişileri işaretleyiniz (birkaç seçenek mümkün olabilir):
   - □ Anne
   - □ Baba
   - □ Benim hepatitis B virüs enfeksiyonum var
   - □ Akraba (örneğin amca, hala)
   - □ Üst tarafta yazılı olan cevapların hiçbiri
   - □ Abi(ler)
   - □ Abla(lar)
   - □ Karım veya hayat arkadaşım
   - □ Bilmiyorum

2. Hiç aile fertlerinden biriyle diş fırçasını paylaştınız mı? (birkaç seçenek mümkün olabilir):
   - □ Evet, birkaç kez (bilmeden veya bilerek)
   - □ Evet, bir defalık (bilmeden veya bilerek)
   - □ Hayır

3. Hiç aile fertlerinden biri ile tırnak makasını paylaştınız mı?
   - □ Evet
   - □ Hayır

4. Hiç aile fertlerinden biri ile traş bıçağı paylaştınız mı?
   - □ Evet
   - □ Hayır

5. Hiç aile fertlerinden biri ile kullanılmış havlu paylaştınız mı?
   - □ Evet
   - □ Hayır

6. Hiç aile fertlerinden biri ile aynı tabaktan yemek yediniz mi?
   - □ Evet
   - □ Hayır

E. Diğerleri

1. Size uyan yeri işaretleyiniz (birkaç seçenek mümkün olabilir):
   □ Türkiyede dövme yaptırdım, piercing taktırdım veya kullaklarımı deldirdim
   □ Belçikada dövme yaptırdım, piercing taktırdım veya kullaklarımı deldirdim
   □ Dövme yaptırmadım, piercing taktırmadım ve kullaklarımı deldirmedim
   □ Üst tarafta yazılı olan cevapların hiçbiri

2. Hiç balıklarla ayak (balık spa) tedavisi oldunuz mu?
   □ Evet, Türkiyede balıklarla ayak tedavisi oldum
   □ Evet, Türkiye dışında başka bir ülkede balıklarla ayak tedavisi oldum
   □ Hayır

3. Babanızın en yüksek derece aldığı diploma?
   □ Diploma almamıştır
   □ Ilkokul diploması (temel eğitim)
   □ Ortaokul diploması
   □ Yüksek okul veya üniversite diploması

4. Annenizin en yüksek derece aldığı diploma?
   □ Diploma almamıştır
   □ Ilkokul diploması (temel eğitim)
   □ Ortaokul diploması
   □ Yüksek okul veya üniversite diploması

5. Hepatit B virüsü aşısı oldunuz mu?
   □ Evet □ Hayır □ Bilmiyorum

   → Cevabınız **Evet** ise kaçtane aşı oldunuz?
   □ 1 □ 2 □ ≥3 □ Bilmiyorum

   → Cevabınız **Hayır** ise neden hepatit B virüsüne karşı aşı yaptırmadınız? (birkaç seçenek mümkün olabilir)

   □ Kendimi iyi hissetiğim için gerek duymadım
   □ Hepatit B virüs aşısından haberdar değildim
   □ Aşı ücretini kendim ödemem gerektiğinden ve pahalı bulduğumdan aşı olmadım
   □ Bilmiyorum
