# Supplementary material for: Early detection of chronic hepatitis B and risk factor assessment in Turkish migrants, Middle Limburg, Belgium
Source: PLoS One. 2020 Jul 27;15(7):e0234740. doi: 10.1371/journal.pone.0234740 (PMC7384618; doi:10.1371/journal.pone.0234740)
Supplement: S6 Fig — (PDF) [file pone.0234740.s006.pdf]

**Name:**…………………………………………
**Surname:**..………………………………….
**Date of birth:**
□□/□□/□□□□
(DD/MM/JJJJ)
**Gender:** female
**Address:**
………………………………………………

**Surname (and name) general practitioner:**
………………………………………………
………………………………………………
………………………………………………
**Region general practitioner:**………………………………….
………………………………………………

QUESTIONNAIRE

A. Demographic information

1. What is your country of birth?
   - □ Belgium → go to question **3**
   - □ Turkey → go to question **2**
   - □ Other → go to question **3**

2. If you are born in Turkey, what year did you come to Belgium? (e.g. the year 1999)

   □□□□

3. Which of your parents were born in Turkey? (multiple choices possible)
   - □ Father → answer question **4**
   - □ Mother → answer question **5**
   - □ None → skip questions **4** and **5**

4. In which region was your father born? (see figure)

□ Region A: Marmara □ Region B: Ege □ Region C: Akdeniz
□ Region D: İç Anadolu □ Region E: Karadeniz □ Region F: Doğu Anadolu
□ Region G: Güneydoğu Anadolu

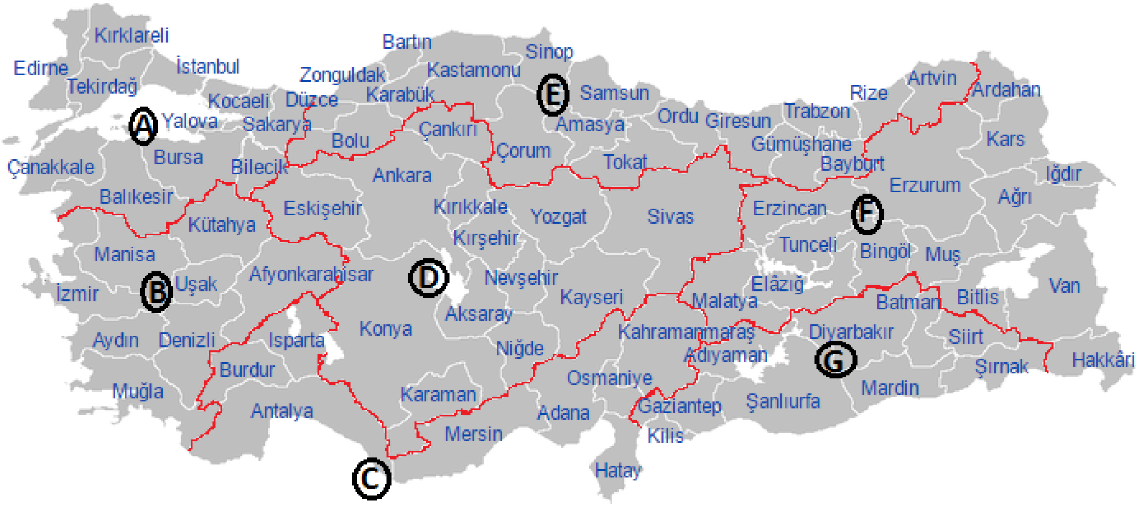

5. In which region was your mother born? (see figure)

□ Region A: Marmara □ Region B: Ege □ Region C: Akdeniz
□ Region D: İç Anadolu □ Region E: Karadeniz □ Region F: Doğu Anadolu
□ Region G: Güneydoğu Anadolu

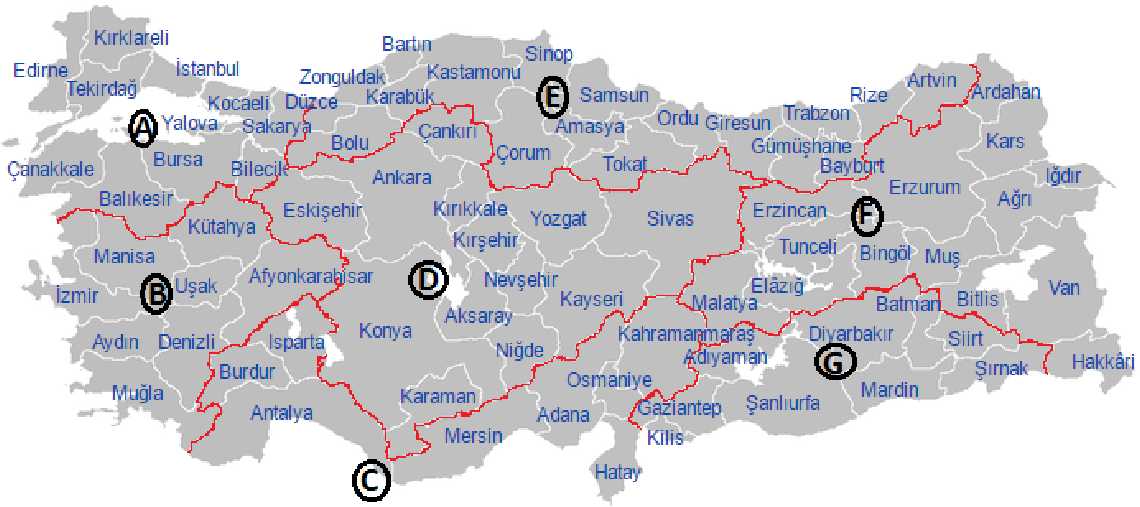

B. Healthcare

1. Have you ever received blood? (multiple choices possible):
   □ Yes, I received blood in Turkey before 1972
   □ Yes, I received blood in Belgium before 1972
   □ Yes, I received blood after 1972
   □ No, I have not received any blood

2. Have you previously been treated at the dentist in Turkey?
   □ Yes
   □ No

3. Have you had a gynaecological examination in the past? (multiple choices possible):
   □ Yes, I have had a gynaecological examination in Turkey
   □ Yes, I have undergone a gynaecological examination in Belgium
   □ No, I have not undergone a gynaecological examination
   □ None of the above answers

4. Have you had surgery in the past? (multiple choices possible):
   □ Yes, I have had surgery in Turkey
   □ Yes, I have had surgery in Belgium
   □ No, I have not had surgery
   □ None of the above answers

5. Have you received treatment with needles (e.g. accupuncture, infusion) in the past? (multiple choices possible):
   □ Yes, I received needle treatment in Turkey
   □ Yes, I received treatment with needles in Belgium
   □ No, I have not received needle treatment
   □ None of the above answers

C. Family

1. Tick which of your family has hepatitis B virus infection (multiple choices possible):
   - □ Mother
   - □ Father
   - □ I have a hepatitis B virus infection
   - □ Other family member (e.g. uncle, aunt)
   - □ None of the above answers
   - □ Brother(s)
   - □ Sister(s)
   - □ Partner
   - □ I don't know

2. Have you ever shared a toothbrush with someone in the family?
   - □ Yes, several times (knowing and/or accidentally)
   - □ Yes, once (knowing and/or accidentally)
   - □ No

3. Have you ever shared a nail clipper with someone in the family?
   - □ Yes
   - □ No

4. Have you ever shared a razor with someone in the family?
   - □ Yes
   - □ No

5. Have you ever shared a used towel with someone in the family?
   - □ Yes
   - □ No

6. Have you ever eaten from the same plate with someone in the family?
   - □ Yes
   - □ No

D. Other

1. Check what applies to you (multiple choices possible):
   □ I have had a tattoo, body piercing or pierced ear in Turkey
   □ I have had a tattoo, body piercing or pierced ear in Belgium
   □ I have not had a tattoo, body piercing or pierced ear
   □ None of the above answers

2. Have you previously received a foot treatment with fish (Fish spa?)
   □ Yes, I got a foot treatment with fish in Turkey
   □ Yes, I got a foot treatment with fish in a country other than Turkey
   □ No, I have not received foot treatment with fish

3. What is your father's highest diploma?
   □ No diploma
   □ Primary school (primary education)
   □ Secondary education
   □ College or university (higher education)

4. What is your mother's highest diploma?
   □ No diploma
   □ Primary school (primary education)
   □ Secondary education
   □ College or university (higher education)

5. Have you been vaccinated against hepatitis B?
   □ Yes □ No □ I don't know
   →If **Yes**, how many vaccinations have you had?
   □ 1 □ 2 □ ≥3 □ I don't know

   →If **No**, why have you not been vaccinated against the hepatitis B virus? (multiple choices possible)
   □ I don't need to be vaccinated because I feel well
   □ I had no knowledge about the possibility of hepatitis B vaccination
   □ I have to pay for the vaccination myself. Vaccine is too expensive
   □ Unknown, I don't know
