# Supplementary material for: Early detection of chronic hepatitis B and risk factor assessment in Turkish migrants, Middle Limburg, Belgium
Source: PLoS One. 2020 Jul 27;15(7):e0234740. doi: 10.1371/journal.pone.0234740 (PMC7384618; doi:10.1371/journal.pone.0234740)
Supplement: S1 Table — (PDF) [file pone.0234740.s007.pdf]

S7 Table. Association of past or recent hepatitis B virus infection to different risk factors among the male study population (n = 468) (weighted GEE model).

Parameter		Estimate (SE)	*p*-value	aOR (95% CI)
(intercept)		-2.38 (0.51)		
Age group			.006	
	40 – 59 years (vs 18 – 39 years)	0.93 (0.40)		2.53 (1.15 – 5.56)
	≥ 60 years (vs 18 – 39 years)	1.64 (0.49)		5.15 (1.96 – 13.58)
Ethnicity			<.001	
	FGM (vs SGM)	1.62 (0.37)		5.05 (2.43 – 10.49)
HBV infected siblings	Yes (vs No/Unknown)	2.35 (0.97)	.022	10.43 (1.55 – 70.24)
Circumcision not carried out by medical doctor	Yes (vs No)	0.98 (0.35)	.004	2.67 (1.34 – 5.35)
Treatment with needles in Turkey	Yes (vs No)	0.63 (0.28)	.030	1.88 (1.08 – 3.28)

Abbreviation: GEE: generalized estimating equations; SE: standard error; aOR: adjusted odds ratio; CI: confidence interval; HBV: hepatitis B virus FGM: first-generation migrants; SGM: second-generation migrants.

First-generation migrants: foreign-born individuals; second-generation migrants: individuals born in Belgium with foreign-born parents.
