# Supplementary material for: Early detection of chronic hepatitis B and risk factor assessment in Turkish migrants, Middle Limburg, Belgium
Source: PLoS One. 2020 Jul 27;15(7):e0234740. doi: 10.1371/journal.pone.0234740 (PMC7384618; doi:10.1371/journal.pone.0234740)
Supplement: S2 Table — (PDF) [file pone.0234740.s008.pdf]

S8 Table. Association of past or recent hepatitis B virus infection to different risk factors among the female study population (n = 623) (weighted GEE model).

**Parameter**		**Estimate (SE)**	***p*-value**	**aOR (95% CI)**
(intercept)		-3.45 (0.33)		
Age group			<.001	
	40 – 59 years (vs 18 – 39 years)	1.04 (0.33)		2.83 (1.48 – 5.41)
	$\geq$ 60 years (vs 18 – 39 years)	1.74 (0.41)		5.71 (2.58 – 12.65)
Ethnicity			<.001	
	FGM (vs SGM)	1.15 (0.34)		3.16 (1.63 – 6.11)
Treatment with needles in Turkey			.003	
	Yes (vs No)	0.75 (0.24)		2.11 (1.32 – 3.36)

Abbreviation: GEE: generalized estimating equations; SE: standard error; aOR: adjusted odds ratio; CI: confidence interval; FGM: first-generation migrants; SGM: second-generation migrants.

First-generation migrants: foreign-born individuals; second-generation migrants: individuals born in Belgium with foreign-born parents.
