# Supplementary material for: Early detection of chronic hepatitis B and risk factor assessment in Turkish migrants, Middle Limburg, Belgium
Source: PLoS One. 2020 Jul 27;15(7):e0234740. doi: 10.1371/journal.pone.0234740 (PMC7384618; doi:10.1371/journal.pone.0234740)
Supplement: S3 Table — (PDF) [file pone.0234740.s009.pdf]

S9 Table. Association of past or recent hepatitis B virus infection to different risk factors among first-generation migrants (n = 628) (weighted GEE model).

**Parameter**		**Estimate (SE)**	***p*-value**	**aOR (95% CI)**
(intercept)		-2.42 (0.32)		
Age group	40 – 59 years (vs 18 – 39 years)<br>60+ years (vs 18 – 39 years)	1.09 (0.31)<br>1.77 (0.34)	<.001	2.99 (1.62 – 5.50)<br>5.84 (3.01 – 11.34)
HBV infected mother	Yes (vs No/Unknown)	0.92 (0.42)	.042	2.51 (1.10 – 5.70)
HBV infected siblings	Yes (vs No/Unknown)	1.05 (0.42)	.024	2.86 (1.26 – 6.49)
Gynaecological examination in Turkey or unsafe circumcision	Yes (vs No/Unknown)	0.64 (0.18)	<.001	1.90 (1.34 – 2.70)
Treatment with needles in Turkey	Yes (vs No/Unknown)	0.57 (0.19)	.003	1.77 (1.23 – 2.54)

Abbreviation: GEE: generalized estimating equations; SE: standard error; aOR: adjusted odds ratio; CI: confidence interval; HBV: hepatitis B virus.

First-generation migrants: foreign-born individuals; unsafe circumcision: collective circumcision and/or circumcision not carried out by medical doctor.
