# Supplementary material for: Early detection of chronic hepatitis B and risk factor assessment in Turkish migrants, Middle Limburg, Belgium
Source: PLoS One. 2020 Jul 27;15(7):e0234740. doi: 10.1371/journal.pone.0234740 (PMC7384618; doi:10.1371/journal.pone.0234740)
Supplement: S4 Table — (PDF) [file pone.0234740.s010.pdf]

S10 Table. Association of past or recent hepatitis B virus infection to different risk factors among second-generation migrants (n = 453) (weighted GEE model).

**Parameter**		**Estimate (SE)**	***p*-value**	**aOR (95% CI)**
(intercept)		-3.75 (0.34)		
HBV infected other family member	Yes (vs No/Unknown)	1.94 (0.54)	.010	6.93 (2.42 – 19.84)
Treatment with needles in Turkey	Yes (vs No/Unknown)	1.22 (0.44)	.030	3.38 (1.43 – 7.97)

Abbreviation: GEE: generalized estimating equations; SE: standard error; aOR: adjusted odds ratio; CI: confidence interval; HBV: hepatitis B virus.

Second-generation migrants: individuals born in Belgium with foreign-born parents.
