# Supplementary material for: Early detection of chronic hepatitis B and risk factor assessment in Turkish migrants, Middle Limburg, Belgium
Source: PLoS One. 2020 Jul 27;15(7):e0234740. doi: 10.1371/journal.pone.0234740 (PMC7384618; doi:10.1371/journal.pone.0234740)
Supplement: S5 Table — (PDF) [file pone.0234740.s011.pdf]

S11 Table. Prevalence of chronic hepatitis B virus infection by different risk factors among the total population (n = 1,081) (weighted univariate GEE).

		N	N	Prevalence (%)	*P* value	Crude OR (95% CI)
Overall		26	1,081	2.4%	-	-
Gender					.046	
Male		18	468	3.8%		2.55 (1.02 – 6.38)
Female		8	613	1.3%		(ref)
Age group					.005	
18-39 years		4	433	0.9%		(ref)
40-59 years		15	511	2.9%		4.28 (1.35 – 13.62)
≥ 60 years		7	137	5.1%		6.14 (1.63 – 23.19)
Ethnicity					.070	
FGM		19	628	3.0%		2.15 (0.86 – 5.39)
SGM		7	453	1.5%		(ref)
Year of immigration (if FGM)					.190	
Before 1987		13	363	3.6%		1.90 (0.86 – 5.39)
Year 1987 or later		6	264	2.3%		(ref)
Southeastern Anatolian origin of mother					.604	
Yes		2	59	3.4%		1.61 (0.37 – 7.00)
No		24	971	2.5%		(ref)
Mother's educational level					-	-
None		15	570	2.6%		
Primary school		10	370	2.7%		

Secondary school		0	123	0.0%		
High school/University		1	13	7.7%		
HBV infected partner					-	-
Yes		0	25	0.0%		
No/Unknown		26	1,056	2.5%		
HBV infected mother					.924	
Yes		1	37	2.7%		1.12 (0.10 – 12.22)
No/Unknown		25	1,044	2.4%		(ref)
HBV infected father					-	-
Yes		0	24	0.0%		
No/Unknown		26	1,057	2.5%		
HBV infected siblings					.025	
Yes		7	45	15.6%		10.57 (2.88 – 38.82)
No/Unknown		19	1,036	1.8%		(ref)
HBV infected other family member					.111	
Yes		5	70	7.1%		4.23 (1.49 – 12.03)
No/Unknown		21	1,011	2.1%		(ref)
Sharing toothbrushes regularly					.090	
Yes		9	188	4.8%		2.52 (1.06 – 5.99)
No		17	892	1.9%		(ref)
Sharing nail clippers					.961	
Yes		24	991	2.4%		1.04 (0.24 – 4.49)
No		2	89	2.2%		(ref)
Sharing razors					.602	
Yes		5	170	2.9%		1.37 (0.46 – 4.06)

No		21	910	2.3%		(ref)
Sharing used towels					.804	
Yes		22	945	2.3%		0.86 (0.28 – 2.69)
No		4	135	3.0%		(ref)
Eating from the same plate at least once					.708	
Yes		23	927	2.5%		1.25 (0.36 – 4.34)
No		3	153	2.0%		(ref)
Gynaecological examination in Turkey (if female)					.896	
Yes		1	99	1.0%		1.10 (0.26 – 4.67)
No		7	514	1.4%		(ref)
Circumcision (if male)					.105	
Collective		8	118	6.8%		2.62 (0.97 – 7.12)
Alone		9	305	3.0%		(ref)
Circumcision not carried out by medical doctor (if male)					-	-
Yes		17	228	7.5%		
No		0	153	0.0%		
Gynaecological examination in Turkey or unsafe circumcision					.001	
Yes		18	330	5.5%		6.93 (2.71 – 17.75)
No		8	751	1.1%		(ref)
Blood transfusion					.845	

Yes		4	167	2.4%		1.12 (0.35 – 3.58)
No		22	914	2.4%		(ref)
Dental treatment in Turkey					.017	
Yes		17	430	4.0%		2.84 (1.23 – 6.60)
No		9	651	1.4%		(ref)
Surgery in Turkey					.183	
Yes		6	133	4.5%		2.30 (0.91 – 5.81)
No		20	948	2.1%		(ref)
Treatment with needles in Turkey					.141	
Yes		11	322	3.4%		2.06 (0.89 – 4.80)
No		15	759	2.0%		(ref)
Body piercing/tattooing/earlobe perforation in Turkey					.038	
Yes		4	327	1.2%		0.38 (0.12 – 1.18)
No		22	754	2.9%		(ref)
Fish spa treatment in Turkey					-	-
Yes		0	64	0.0%		
No		26	1,017	2.6%		

Abbreviation: OR: odds ratio; CI: confidence interval; FGM: first-generation migrants; SGM: second-generation migrants.

First-generation migrants: foreign-born individuals; second-generation migrants: individuals born in Belgium with foreign-born parents; unsafe circumcision: collective circumcision and/or circumcision not carried out by medical doctor.
