# Supplementary material for: Early detection of chronic hepatitis B and risk factor assessment in Turkish migrants, Middle Limburg, Belgium
Source: PLoS One. 2020 Jul 27;15(7):e0234740. doi: 10.1371/journal.pone.0234740 (PMC7384618; doi:10.1371/journal.pone.0234740)
Supplement: S6 Table — (PDF) [file pone.0234740.s012.pdf]

S12 Table. Vaccination status (solely anti-HBs positive) by different risk factors among the total population with information on anti-HBs (n = 1,077) (weighted univariate GEE).

	N	N	Prevalence (%)	*P* value	Crude OR (95% CI)
Overall	247	1,077	22.9%	-	-
Gender				.006	
Male	84	466	18.0%		0.67 (0.50 – 0.89)
Female	163	611	26.7%		(ref)
Age group				<.001	
18-39 years	195	432	45.1%		(ref)
40-59 years	47	509	9.2%		0.12 (0.08 – 0.17)
≥ 60 years	5	136	3.7%		0.04 (0.02 – 0.11)
Ethnicity				<.001	
FGM	54	626	8.6%		0.13 (0.09 – 0.18)
SGM	193	451	42.8%		(ref)
Year of immigration (if FGM)				.005	
Before 1987	22	361	6.1%		0.38 (0.21 – 0.70)
Year 1987 or later	32	264	12.1%		(ref)
Mother's educational level				<.001	
None	67	567	11.8%		0.05 (0.02 – 0.18)
Primary school	102	369	27.6%		0.13 (0.04 – 0.46)
Secondary school	68	123	55.3%		0.40 (0.11 – 1.43)
High school/University	9	13	69.2%		(ref)
Vaccination history				<.001	

Not vaccinated/Unknown	108	837	12.9%		0.11 (0.08 – 0.15)
Vaccinated	139	239	58.2%		(ref)

Abbreviation: OR: odds ratio; CI: confidence interval; FGM: first-generation migrants; SGM: second-generation migrants.

First-generation migrants: foreign-born individuals; second-generation migrants: individuals born in Belgium with foreign-born parents;

unsafe circumcision: collective circumcision and/or circumcision not carried out by medical doctor.
